# Mental Health Prescribers’ Perceptions on the Use of Pharmacogenetic Testing in the Management of Depression in the Middle East and North Africa Region

**DOI:** 10.2147/PGPM.S410240

**Published:** 2023-05-30

**Authors:** Shimaa Aboelbaha, Monica Zolezzi, Oraib Abdallah, Yassin Eltorki

**Affiliations:** 1 College of Pharmacy, QU Health, Qatar University, Doha, Qatar; 2 Clinical Pharmacy and Practice, College of Pharmacy, QU Health, Qatar University, Doha, Qatar; 3 Mental Health Service, Hamad Medical Corporation, Doha, Qatar

**Keywords:** pharmacogenetic testing, depression, qualitative study, MENA region, psychiatry, mental health

## Abstract

**Objective:**

A wide variety of commercial pharmacogenetic (PGx) tools are available worldwide to guide treatment selection for depression based on individuals’ genetic profiles. However, the use of genetic testing to inform psychiatric care has faced challenges due to the limited training and education for mental health clinicians. The aim of this study was to explore the knowledge, level of engagement, and perspectives on the use of PGx testing when making depression management decisions among practicing psychiatrists within the Middle East and North Africa (MENA) region.

**Methods:**

This is a qualitative study using semi-structured interviews. Consenting psychiatrists were interviewed through an online platform (Skype^TM^ or Microsoft Teams^TM^). Interviews were audio recorded, transcribed, and thematically analyzed with the assistance of NVivo^®^ software.

**Results:**

Eighteen interviews from 12 countries have been conducted. Analysis of the current interviews produced five major themes including: (1) Overall perceptions and attitudes; (2) Knowledge and awareness; (3) Education, training, and professional experience; (4) Facilitators and barriers; and (5) Ethical dilemmas. These themes support the notion that there is limited, mostly basic, education, knowledge, and training regarding genetic testing in the management of depression, although there is significant interest and willingness in the part of prescribers to adopt this strategy in their practice.

**Conclusion:**

The findings of the study suggest that psychiatrists practicing in the MENA region appear to be interested in implementing PGx testing when managing people with depression. However, it is also important to recognize that this cannot be achieved unless more supporting strategies are implemented within their current health system environment.

## Introduction

Globally, depression ranks among the most prevalent mood disorders.^1^ It is considered one of the leading causes of disability and a major contributor to the global burden of disease.^1^ According to the World Health Organization (WHO), more than 300 million people are currently living with depression.^2^ The incidence of depression has almost doubled over the past 20 years from 172 million cases in 1990 to 258 million cases in 2017.^3^ The number of incident cases of depression in the Middle East and North Africa (MENA) region was reported to be 26 million cases in 2017.^3^ The increase in incident cases of depression in Qatar has been reported as the highest in the MENA region with more than a 5-fold increase between 1990 and 2017.^3^ According to the Qatar Ministry of Public Health (MoPH), 25% of people who approached primary care services were affected with at least one mental disorder, of which 13.5% had depression.^4^

The ultimate goal of treating depression is achieving remission whereby no depressive symptoms affect a person’s quality of life, with the least possible adverse effects. Studies have shown that antidepressants (ADs) provide significantly better clinical outcomes than placebo, especially in the more severe forms of the disease.^5^ However, the majority of patients fail to achieve remission.^6–8^ The Sequenced Treatment Alternatives to Relieve Depression (STAR*D) trial has shown that only 30% of patients were able to achieve remission following treatment with selective serotonin reuptake inhibitors (SSRIs).^6^ In a European study, more than 50% of patients with depression showed resistance to treatment after two AD trials at adequate doses and duration.^9^ Research has been indicative that this variability in responding to AD treatment may be related to genetic predisposition that affects patients’ ability to metabolize medications or change the intensity of response exerted by these medications.^10^,^11^ For example, patients who are known to be CYP2D6 poor metabolizers may have higher concentrations of medications that are metabolized through the CYP2D6 enzyme system, and thus may be at greater risk of experiencing adverse effects or show better response as compared to rapid metabolizers.^12^ The science that addresses these issues is called pharmacogenetics (PGx) which is defined as the study of differences in response to medications due to differences in the genetic makeup of individuals.^13^ A genome wide association study (GWAS) of a sample of 2799 people who received treatment for depression showed that common genetic variants contributed to 42% of the differences seen in their response to ADs.^14^ As such, over the past few years research has been directed to explore the impact of PGx when applied in clinical practice. This is achieved through the use of PGx tests. These are a type of genetic tests that analyze patients’ DNA from a blood or saliva samples to identify specific genetic variations that may affect their response to medications. The results are usually reported in a format presented to healthcare providers, who can then use the information to make informed treatment decisions.^15^ PGx tests are different from normal genetic tests in a way that the latter look for a wide range of genetic variations that may be associated with certain health conditions or traits. They may be also used to determine paternity or ancestry. Additionally, normal genetic tests are not specific to medications and are rarely used to guide treatment decisions.^16^

The results of a systematic review of systematic reviews on PGx-guided treatment with ADs showed higher response and remission rates than in the control groups, particularly in the more severe forms of depression.^17^ The most recent meta-analyses conducted on this topic revealed statistical significance in favor of PGx testing in improving response and remission to ADs as compared to traditional treatment.^18–20^

Despite these advances, the uptake of genetic testing in psychiatry has progressed at a slower pace than in other fields such as cardiology and cancer settings, with the implementation of PGx-guided management strategies even lacking in some regions of the world.^21^ It has been suggested that PGx adoption by healthcare providers’ (HCPs) is challenging due to lack of preparedness, knowledge and experience in this area, possibly attributed to the rapid progress in this field.^22^ Other reasons include the complex nature of psychiatric conditions, lack of definitive evidence, limited availability of counselling services, and lack of reimbursement plans.^23^ Vest et al has reported that primary care and mental health providers from six healthcare sites in the United States (U.S) demonstrated limited experience and knowledge of this concept particularly for psychiatric medications.^24^ Additionally, it was found that PGx application in psychiatry is further challenged by the high cost of testing and the lack of cost effectiveness studies as reported by a previous research to assess concerns of HCPs regarding PGx testing in depression.^22^,^25^

Several studies were conducted over the past decade to assess HCPs’ perceptions and attitudes towards genetic testing. The majority of these studies were conducted in Europe and the U.S. A previous study involving 194 physicians and pharmacists in mental health highlighted that 46% of participants felt confident to use these tests.^26^ Another study involved pharmacists practicing in psychiatric settings viewed PGx testing as a useful tool, however, only 36% considered themselves knowledgeable about the concept.^27^ Only a few studies with this scope were conducted in the MENA region.^28–31^ These studies were based on single countries only and no study attempted to gain insight into all countries within the region. Additionally, the studies available were conducted in settings other than psychiatry. Therefore, this study was conducted with the aim of exploring the knowledge, level of engagement, and perspectives on the use of PGx testing when making depression management decisions among practicing psychiatrists within the MENA region.

## Methodology

### Study Design

This was a qualitative study using semi-structured interviews of key informants to explore viewpoints of psychiatrists working in mental health settings in the MENA region.

### Study Population

Practicing psychiatrists from the MENA region countries willing to participate in the study, and fluent in either Arabic or English were eligible to be included in the study. We aimed to recruit at least one psychiatrist from each country in the MENA region.

### Data Collection

Convenience sampling was used by study team members (YE, OA) who, at the time of the study, were affiliated with the Mental Health Hospital (MHH) in Qatar. They identified potential participants who fulfilled the eligibility criteria and provided names and contact details. Snowball sampling was then applied when participants who completed the interviews were asked to nominate other participants within their practice or colleagues who would meet the eligibility criteria.

All nominated participants were initially contacted via email to introduce them to the study and ask if they were willing to participate. Two email/message reminders were sent 10 days apart after the first contact. After participants’ initial response, their eligibility in the study was reconfirmed by the study investigator. Then, they were given a brief description about the study title, objectives, and procedures. Consent forms were sent via email, and they were asked to review, sign, and send them back before the interview. Then the interviews were scheduled at a mutually convenient time and took place either online via platforms such as Microsoft Teams, Skype, or Zoom, or face to face (in case of participants from Qatar) in the MHH. The interviews lasted from 15 to 20 minutes and each interview involved only the interviewer (SA, BSc (Pharm), MSc) and participants. At the time of the study, the interviewer was a female research coordinator who had no established relationships with any of the participants. Additionally, she had received extensive training in qualitative research. An overview of the study objectives was presented at the beginning of each interview, and participants were informed that they may withdraw from the study at any time during or after the interview. All interviews were audio recorded and the audio recordings were transcribed verbatim and analyzed using NVivo^®^ software. Data collection continued until saturation of themes was reached. Saturation of data was determined when analysis of new interviews yielded no new information or insights after the 13th interview.^32^ This is in line with the recommended sample size for saturation in qualitative studies.^33^ All the interviews were conducted between November 2021 and September 2022.

### Interview Guide

An interview guide consisted of a set of pre-formulated questions was developed through discussion and iterative refinement by the research team, to elicit information from participants on the following domains:

- Demographics, including age, gender, nationality, country of practice, years of practice in psychiatry, and professional title.
- Professional experience with PGx testing in the management of depression and mental disorders in general.
- Awareness, knowledge, and education about genetics and PGx in the field of psychiatry.
- Perceptions and beliefs about the use of PGx in the treatment of depression and mental disorders.
- Previous training related to genetics and perceived training needs of HCPs to incorporate PGx into clinical practice.
- Perceived facilitators and barriers to the implementation of PGx into clinical practice.
- Ethical considerations related to genetic testing and psychiatry.

With one exception, all the interviews were conducted in English. The single interview that was conducted in Arabic was translated by two native Arabic speakers from the research team (SA and OA) who were also fluent in English.

### Data Analysis

Demographics of participants were summarized using descriptive statistics. Inductive thematic analysis was used as the study aimed to capture the diverse perspectives and experiences of participants on the use of PGx testing. Verbatim transcripts of taped interviews were produced by SA, and initial codes were produced by two investigators (SA and OA). The codes were then categorized and collapsed into themes using NVivo^®^ software. The themes and subthemes were then reviewed and checked for validity by two study investigators (OA and MZ) to identify the synergies, points of difference, and reach consensus.

## Results

### Participants’ Characteristics

Table 1 represents participants’ demographic characteristics. The majority of participants were males. Participants were enrolled from 12 countries from the MENA region, including Qatar, Kuwait, Oman, Saudi Arabia, United Arab Emirates (UAE), Bahrain, Egypt, Jordan, Iraq, Palestine, Tunisia, and Algeria. At the time of the interviews, the majority of participants had more than 15 years of experience in psychiatry, and more than half held senior consultant degrees. Table 2 summarizes the five major themes and subthemes that emerged from the interviews. The major themes and subthemes are described in details below.

**Table 1:** Participants’ Characteristics

Characteristics	N (%)
**Gender**	
Male	13 (72.2)
Female	5 (27.8)
**Age**	
30–40	6 (33.4)
41–50	2 (11)
51–60	7 (38.9)
>60	3 (16.7)
**Country of practice**	
Qatar	4 (22)
Saudi Arabia	3 (16.7)
Bahrain	2 (11)
Jordan	1 (5.6)
Egypt	1 (5.6)
Tunisia	1 (5.6)
Oman	1 (5.6)
Palestine	1 (5.6)
Iraq	1 (5.6)
Kuwait	1 (5.6)
United Arab Emirates	1 (5.6)
Algeria	1 (5.6)
**Degree**	
Senior consultant	12 (66.6)
Specialist	3 (16.7)
Assistant professor with board certification	1 (5.6)
Physician	1 (5.6)
Senior registrar	1 (5.6)
**Years of practicing psychiatry**	
<5	2 (11)
6-15	4 (22)
16–25	6 (33.4)
>25	6 (33.4)
**Settings**	
Mixed settings	15 (83.3)
Outpatient	3 (16.7)

**Table 2:** Major Overarching Themes

Themes	Subthemes
**1. Overall perceptions and attitudes**	1a. Enabling perceptions and attitudes 1b. Disabling perceptions and attitudes 1c. Indications to request PGx testing
**2. Knowledge and awareness**	2a. Overall good awareness 2b. Overall basic knowledge
**3. Education, training, and professional experience**	3a. Limited education, training, and professional experience 3b. Qualifications and training needs
**4. Facilitators and barriers**	4a. Facilitators for implementing PGx 4b. Barriers for the uptake of PGx strategies
**5. Ethical dilemmas**	5a. Informed consent and confidentiality 5b. Transparency

### Major Overarching Themes

Five major themes emerged from the one-to-one semi-structured interviews, namely overall perceptions and attitudes, knowledge and awareness, education and professional experience, facilitators and barriers, and ethical dilemmas.

### Theme 1: Overall Perceptions and Attitudes

Several perceptions and attitudes towards PGx testing and its application in the management of psychiatric disorders and depression emerged from the psychiatrists’ interviews, which were grouped into three subthemes: Enabling perceptions and attitudes, disabling perceptions and attitudes, and perceived indications to request PGx testing.

#### Subtheme 1: Enabling Perceptions and Attitudes

Participants perceived the incorporation of PGx testing into clinical practice as a useful and promising strategy, which is better than the traditional “trial and error” approach that the participants described being used currently in the management of depression. On view of the many advantages of precision medicine, participants also indicated that it would become the future practice of medicine.

As a potential I think it is amazing and I think it is the future direction and we should push for it because as at the moment we are just relying on trial and error, and it is not fair to the patients. (P02)

Participants also perceived that PGx test reports provide a straightforward, standardized and relatively simple to use tool, which some described as being similar to the ones used for culture and sensitivity reports for choosing the most appropriate antibiotic.

I mean, like the reports, I think, it will be self-explanatory, like this area needs this, this works like this. I believe the result will be something like antibiotic resistance. This is my imagination. (P03)

It will be the way I think about it is like the treatment with the cultural sensitivity test we do for people who have bacterial infection. So, we grow the bacteria, and we subject it to the different antibiotics and then we can check which one is sensitive. (P06)

A variety of possible advantages for the use of this approach were also highlighted by the participants, such as: saving time and reducing cost of illness, improving patients’ response, decreasing side effects, and reducing the misconceptions that psychiatry practice is less science-based than other medical disciplines.

We will save much time and effort trying different medications until we find the one suitable for him. This process can take years for some patients, and it have lots of effect on the lives and lifestyle. (P03)

Also, I think it might -and this is like another personal point of view- it might make psychiatry, less stigmatized,psychiatry is very heavily stigmatized nowadays by our colleagues from other specialties. At least it may make psychiatry sound more scientific to them. (P09)

#### Subtheme 2: Disabling Perceptions and Attitudes Towards PGx Testing

Participants also described some concerns, mostly disabling or displaying a negative attitude, towards the use of PGx testing in mental health. For the most part, these concerns were displayed as fears that these genetic tests would replace clinical judgement, harm the patient-doctor relationship, and disregard the value of clinical experience at the time of decision-making.

On the other side, maybe it will make us subside our clinical expertise and clinical practice because when we depend on machines, we put aside some of our clinical experience. (P014)

At the moment, we are using our clinical sense in terms of selecting antidepressant we talk about side effect profile, we talk about other contraindications, especially if a person is having a medical comorbidity which antidepressant might worsen. So, it involves a human or doctors’ skills and experience in the process. So, the disadvantage, it might take away the part of psychiatry or psychopharmacology. (P06)

There were mixed responses regarding whether the changes made to patients’ therapy based on these reports have generated positive clinical outcomes.

Currently, from the samples that I have seen. None of them was useful. None of them I found that informing me regarding the right direction and like I said, the reports, although they were from three different centers but the way it was written, if I recall, was kind of, possibility of, likelihood of, it was all written in that way and most of the medication was the same in terms of possibility of side effects like that report so it was not that useful. (P02)

I’ve seen a good number of these patients and a good number of these tests. I would say in my case, I had 90% improved. I had only two unfortunate cases, those two cases, one of them I missed in the follow up and the other one just stopped the management plan thinking that like this is enough for me. (P10)

#### Subtheme 3: Indications to Request PGx Testing

There appeared to be an overall perception that, in view of the current evidence regarding its clinical utility not being robust enough, and because of the high costs associated with their use, reserving these tests for treatment resistant cases would be the preferred indication.

I think logically, I would refer them once they are resistant, not like first line given that the evidence might not be as robust now and given also the cost I mean, the cost if you do it for everyone maybe it will drive the costs up. (P09)

I think I will reserve it to the resistance because of the cost. Because we work in general psychiatry, and we do not have so much money for that. (P08)

Usually, when we have resistant depression, we look for causes. One of it is, you know, the genetic testing that the specialist set up for medicine. So, it is not a routine just for a resistant case. (P11)

### Theme 2: Knowledge and Awareness

Participants’ overall knowledge and awareness about the application of PGx in the context of mental illness, and more specifically in depression management, also emerged as a major theme. Under this theme, two subthemes emerged, namely overall good awareness, and overall basic knowledge.

#### Subtheme 1: Overall Good Awareness

Participants’ disclosures that were mostly describing their awareness rather than their scientific knowledge were grouped under this subtheme. For the most part, participants perceived themselves as having an overall good background about the main concept of PGx and precision medicine, as well as about the emerging role of PGx testing in the field of psychiatry. They also indicated to be aware that PGx testing was not yet fully adopted in practice which was predominantly described as being due to the lack of robust clinical utility studies. They also reported being aware of the role of the variations in pharmacokinetic and pharmacodynamic genes in predicting response and side effects to medications. Also, they mentioned that these differences prompt modifications to patients’ therapy in a more personalized manner, in order to achieve the best possible outcomes.

Basically, that the genetic testing, allow us to know the difference between the fast metabolizer, slow metabolizer and determines what dosage would be appropriate for different patients based on research, they can tell us which genetics will respond to which antidepressant or antipsychotics and which one will have side effects. (P15)

I know it is evolving. I am reading about it in the American Journal that they are doing it and it is more specific to choose the medication, it is better than trial and error method that we are using. (P07)

I know there is genetic difference between groups and even individual in terms of response to different medications (…) there are certain medications that does not give any effect only give side effects and there’s also lots of research on this area. Part of this topic also is for the titration and Cytochrome P450 and its subtypes it shows how the drug is metabolized (…) According to this we will have to adjust the dose; up it or lower it and also. (P03)

#### Subtheme 2: Overall Basic Knowledge

Participants’ disclosures that were mostly describing their scientific and evidence-based knowledge rather than describing overall awareness about PGx testing in mental health practice were grouped under this subtheme. For the most part, participants were unaware about PGx testing guidelines or of similar resources that provide evidence-based clinical recommendations according to genetics data.

I do not think that there is a guideline yet but there are researchers in the field. I do not know. But I think there is no guidelines in that. (P08)

I went through literature, primary literature not like getting back to guidelines per se, I do not know about any. (P18)

When asked about combinatorial PGx tests, participants reported limited knowledge about what this term refers to. Although some indicated to have an overall awareness on this topic, they also indicated to have limited understanding of this concept.

I knew it. I just did not know the name. (P10)

I heard about genetic panels in the U.S. but honestly, I do not know exactly the name. (P09)

Participants also reported being aware that genetic testing is safe, however, it has controversial efficacy as more robust evidence is still required.

For safety yes. I think it is safe. For efficacy I mean it is controversial. (P03)

The last I read is that there is big gap between one report and the other. I had two views, one of them was saying that it is only 30% effective and one of them was saying something like 84%. (P10)

I know it is safe there is no harm with them, but I am not sure of how the robust the evidence is about their efficacy. (P16)

### Theme 3: Education, Training and Professional Experience

Participants’ previous genetic-related education and training, and their clinical professional experience with the application of PGx tools were also explored. Under this major theme, their responses generated two subthemes, namely limited education, training and professional experience, and qualifications and training needs.

#### Subtheme 1: Limited Education, Training and Professional Experience

For the most part, participants reported not having any professional education or specific training on PGx, particularly during their medical school. However, attendance to seminars, conference presentations, or continuing professional development (CPD) workshops were reported by some participants.

Really no you know, because not available in the Middle East areas. (P12)

Only basic knowledge and training after graduation. (P16)

Once there was a drug company who came, I mean, not the lab, a lab from Spain who came and brought up speaker. So, it was a CPD, I would say. (P15)

I attended lectures about it and workshops. (P18)

In addition to the lack of training and education, participants also reported having limited clinical experience with genetic testing.

In my practice, I did not read any report or done any, any genetic referral. (P07)

Some participants working in governmental institutions or in the private sector in countries belonging to the Gulf Cooperation Council (GCC) reported having counselling experience with test reports that their patients brought from abroad.

We use local tests here. We usually use… I forgot the name, but we use this one and I think recently, we had another one that has been conducted or analyzed in Dubai. (P10)

They do it outside of course, they do them in Europe, maybe in the States then they come with it accompanied by a report from Canada like that. We do not ask them to do that, who ask them is in the private practice. (P14)

#### Subtheme 2: Qualifications and Training Needs

Participants suggested the application of PGx into psychiatric practice requires additional qualifications and training. They also provided a variety of suggestions to enhance the implementation of PGx testing in psychiatry within the MENA region. For the most part, participants believed psychiatrists should hold at least a senior specialist or a consultant status in order to have the authority to order these tests.

I think the physician who will request this should be at least a consultant or in a tertiary psychiatric facility, I do not think it will be suitable for a primary care physician, for example, to refer a patient for such test. (P12)

A least the senior specialist or consultant. (P06)

Participants predominantly from GCC also pointed at the role of pharmacists in facilitating the application of PGx into practice. Their personal working experience has provided them with exposures to pharmacists who were able to be involved in interpreting the PGx results, counselling patients, and even leading training workshops on how to integrate this approach into practice settings.

I think pharmacist as a colleague, and as a coworker with us, is very much needed in this domain. (P14)

May be better for the pharmacy to train or to lead the training project across the country, you know, for training clinicians in mental health. (P05)

I would say both should be trained, the psychiatrist and the pharmacist, should be trained to do this, not only one category, so they can help each other. (P18)

Participants suggested training options that they believe will be beneficial in the integration of PGx testing into clinical practice. The options suggested were divided into two categories: formal training, and CPD courses. The formal training included master’s degree in genetics and PGx training during residency program.

Residents or board-certified doctor can do a master and the training in hospital in genetic department. Both. So, master’s in the university with training in genetic departments to know how to interpret the tests. (P08)

I think that you would need to incorporate information and theories into training about how to interpret the tests, when to request them. I think they should be incorporated into the residency program for psychiatry residents. (P09)

For CPD, participants suggested the implementation of postgraduate seminars, webinars, conference sessions, and online courses.

I would prefer, I do not think that this even exists, but I would prefer an online course at least from Harvard or something like this on the interpretation of the results. (P03)

Presentations, webinars, and then you take from time to time, small group of them, do an in vivo training for them just to teach them on case-to-case basis what was the issue and how did we solve it and why and how did we fail to solve it. (P12)

### Theme 4: Facilitators and Barriers

Under this main theme, participants’ opinions on how the implementation of PGx-guided therapy into routine psychiatry practice could be facilitated were explored, as well as the factors they considered as important barriers. Their opinions were grouped in two sub-themes: facilitators for implementing PGx, and barriers for the uptake of PGx strategies in the MENA region.

#### Subtheme 1: Facilitators for Implementing PGx

The availability of resources such as genetic tests, specialized laboratories for the analysis of patients’ samples, and clinical practice guidelines were predominantly identified by participants as facilitators. Some participants also identified the availability of training for clinicians, having a quick return time of results, and increased patients’ awareness on PGx-guided options as facilitators.

So, facilitation is probably availability of the test, having a clear criterion of when to use it. (P06)

Implementation of genetically based guidelines in clinical practice. More conferences about this issue, and most importantly, the availability of the test and how can I refer the patient and to where I refer the patient, the labs and availability of the tests in there. (P12)

I think a facilitator would be making it easier to request these lab tests, having a quicker return time because like I was saying earlier, you do not want to keep the patient without medication, while they have severe depression or even moderate depression, especially in the absence of alternative support, and we’re having to rely on medication. (P02)

#### Subtheme 2: Barriers for the Uptake of PGx Strategies in the MENA Region

Participants brought forward a variety of barriers that might stand against the implementation of this approach in the MENA region. The high cost of the tests and the lack of insurance support emerged as a prominent barrier. Other barriers that were identified include lack of resources and education.

I think that the cost. In my opinion, I think the cost is the biggest obstacle we are having with these tests. (P10)

In my country, the cost will be the main thing that I do not see for the next 10 years. It will be very expensive here. (P07)

### Theme 5: Ethical Dilemmas

This major theme emerged from respondents’ ethical-related concerns and implications associated with the implementation of PGx testing into routine psychiatric practice. Several safeguards emerged under this theme and grouped into two subthemes: informed consent and confidentiality, and transparency.

#### Subtheme 1: Informed Consent and Confidentiality

Participants perceived that taking patient’s consent is very important and it could be a major ethical dilemma that might arise during the implementation of this approach into practice. It was highlighted by participants that patients should clearly understand the purpose of these tests and provide consent for using them for the management of their disease. They also highlighted that patients need to be ensured that their data will not be shared or used for any other purpose than for improving their care.

The most important thing is for the patient to agree. You know we still have the stereotyping for the mental illness so not everyone would agree. (P01)

Well, if the patient consents, and there is, I think there is no harm ethically or legally in this one. So, I think written consent will help move the process. (P03)

#### Subtheme 2: Transparency

Participants highlighted the need to ensure that complete disclosure is provided to patients about what and how information from genetic testing will be used. They highlighted the importance of being transparent with their patients in terms of interpreting their genetic data and disclosing any conflict of interest to ensure that patient’s trust is not compromised.

Well, this is a very shady area, because you can present them as a very good solution and probably a bypass, saving you plenty of time and money but at the same time, if you were not open about discussing the cost, and probably false negative results, false positive, you will be probably misusing your patient trust. (P10)

The ethical issue would be the concept of are we telling the truth to the patient? Is this just as predictable? Is there an element of advertising and making profits for drug companies? … and there is always this concept of the role relationship between doctors and pharma, so it might bias in the process of prescribing or practicing psychopharmacology. (P09)

## Discussion

This study helped shed light on psychiatrists’ perceptions on the use of PGx in the management of mental health conditions, particularly depression within the MENA region. To our knowledge, there are few country-specific studies from the Middle East that assessed perceptions and beliefs of HCPs and students towards PGx in general.^28–31^ However, for the most part, these studies were conducted in single countries, or included participants who were not directly involved in prescribing medications to treat mental health conditions. Therefore, this study adds value to the literature as it explores the readiness of the current health systems within the MENA region to practice precision medicine, especially in mental health.

Overall, psychiatrists viewed genetic testing as a very promising approach that might be a future solution to the treatment challenges currently faced in regard to depression management. These positive perceptions were related to the benefits that precision medicine provides, suggesting that participants are inclined to practice this approach and are aware of its advantages. This finding was consistent with the results from similar studies within the MENA region, Malaysia, and Canada.^28^,^34–36^ Participants also highlighted important patient-related advantages to the use of PGx testing including improving patients’ response to ADs treatment and their quality of life, which is also in concordance with findings from previous clinical utility studies of combinatorial genetic testing in patients with depression.^37^,^38^ On the other hand, few participants expressed concerns related to PGx implementation as they viewed it as a threat to their career and that it may replace their clinical expertise. However, PGx is not meant to replace clinical expertise but rather to complement it as it provides HCPs with additional information that can help guide treatment decisions.^39^

Another interesting finding that was highlighted during the interviews is the general underlined misconception surrounding psychiatry practice as not being evidence-based, and even being regarded as a pseudoscience by other medical professions. Participants highlighted that this misconception may be responsible for medical students turning away from the field of psychiatry, and the reduced number of postgraduate training programs available, leading to a general shortage of psychiatrists.^40–42^ A number of studies have shown that healthcare students and professionals lack enthusiasm for psychiatry due to their belief that there is limited evidence supporting treatment choices, difficulty measuring treatment efficacy, and poor prognosis for those treated with mental illness.^40–43^ Participants in the current study believed that the use of PGx would enable psychiatry to become less subjective and might help to correct previous misconceptions regarding mental health treatments. For example, participants viewed the reports generated from genetic testing as having the same scientific value as those that are generated by antimicrobial resistance tests. Although these perceptions could be viewed as positive, it is worth mentioning that these reports lack standardization possibly due to the large number of manufacturing companies producing these tests, and the variety of methods utilized in data integration and how the results are presented to users.^44^ In order to address this issue, the Clinical Pharmacogenetics Implementation Consortium (CPIC), the Dutch Pharmacogenetics Working Group (DPWG), Centers for Disease Control and Prevention (CDC) PGx workgroup, and PGx information list issued by the U.S Food and Drug Administration (FDA) aimed at facilitating a more uniform implementation of PGx. This is achieved through publishing recommendations to guide the use of standardized terminology and genetic variant nomenclature across different tests. In addition, it includes clinical guidelines for interpreting and implementing PGx testing results into practice.^45–49^

Another remarkable finding is that, for the most part, psychiatrists in the MENA region have limited experience requesting genetic testing for their patients. This is in part due to the limited availability of such tests in their countries, the high cost of the service, and lack of insurance coverage. This was found in concordance with findings from previous international studies.^24^,^34^ Participants from countries within the GCC such as Qatar, Saudi Arabia and the UAE, were the only ones who reported having previous experience ordering PGx tests and providing counselling based on their results, although these were mostly within the private sector where patients were required to pay. These results reflect a faster implementation of precision medicine in high-income countries as compared to low-to-middle-income countries within the MENA region. Therefore, it is likely that financial challenges are the primary barrier to the wider implementation of PGx testing rather than healthcare professionals’ unwillingness or resistance. This financial barrier was also highlighted in other studies from different geographical regions across the world, indicating that it is not unique to the MENA region.^31^,^50–52^ Despite the high cost of these interventions, cost effective analyses have shown that PGx-guided treatment represents a cost saving option.^53–55^ Furthermore, the application of genetically-guided treatment in psychiatric outpatient settings in the U.S resulted in reduced healthcare costs over a 4-month follow up period.^56^ Participants of a study to explore attitudes and interests of stakeholders towards PGx implementation in the UAE proposed a solution to this barrier which entails building a country-based internal infrastructure that facilitates processing patients’ samples sent for genetic testing. Stakeholders suggested that this approach should help in reducing the cost, maintaining privacy, and avoiding delays in receiving the results.^29^

Other studies reported on other barriers that were not described by participants in our study. For example, barriers were in relation to fitting time during practice to discuss test results with patients, and misinterpretation of the data under the color-coded categories that are used in the tests reports.^24^,^31^ There is a possibility that these barriers were not addressed in the present study as the majority of participants did not have actual encounters with tests reports, and therefore lacked knowledge on whether data interpretation would be challenging or time consuming.

Despite the good awareness reported by our study participants, their knowledge of some important PGx terminology, such as combinatorial PGx tests, or relevant clinical practice guidelines for the use of PGx, was limited. This finding could be attributed to the lack of clinical experience with PGx amongst most of our participants, as well as lack of previous training and education in PGx. However, they acknowledged the importance of integrating more courses on this topic into educational curricula for pre- and post-graduate healthcare students as well as holding CPD activities focusing on this area. This also emphasizes the positive attitudes towards PGx observed during the interviews. Similarly, healthcare workers from previous studies have shown lack of confidence in the use of PGx test results which was attributed mainly to their lack of knowledge and training.^28^,^31^,^57^,^58^ Participants who received prior training, on the other hand, had a significant improvement in PGx knowledge scores.^59^ This further supports the need for integrative PGx-based educational programs and courses since raising HCPs’ awareness is considered a key factor to facilitate the diffusion of PGx into practice.

Participants from the GCC have emphasized the important role of pharmacists in facilitating the implementation of PGx into routine care. This brings into focus two major aspects: firstly, the high awareness by doctors practicing within the GCC of the role of pharmacists in the mental healthcare team; and secondly, the value that pharmacists might add in enhancing the integration of PGx programs into health systems within the Middle East. Pharmacists play a dynamic role in ensuring safe and effective medications adminstration for patients through their regular interactions with HCPs, patients, and caregivers. They also have a promising potential to impact healthcare systems about the significance of PGx through their capacity in guiding medication selection and dosage adjustment based on PGx tests outcomes.^60^ Furthermore, the integration of PGx counselling services by clinical pharmacists along with medication therapy management (MTM) services could result in more favorable patient outcomes.^61^ A recent survey conducted at the MHH in Qatar to assess physicians’ and nurses’ perceptions of clinical pharmacists’ services revealed that both professions highly valued the role of pharmacists in managing patients’ regimens, monitoring for efficacy of medications, and addressing adherence.^35^ Another national survey study from Qatar explored the awareness and attitudes of HCPs toward the application of PGx highlighted that both physicians and pharmacists had a similar level of PGx awareness. Furthermore, pharmacists perceived themselves responsible for all tasks involved with genetic testing. Also, they demonstrated a more positive attitudes and beliefs towards the benefits of genetic testing as compared to physicians.^28^ These findings were also supported by data from the UAE.^62^,^63^ Another survey study aiming at assessing clinician’s perceptions of PGx use in psychiatry was conducted in Singapore. Results showed that pharmacists, as compared to psychiatrists, felt more competent in recommending the testing service, identifying situations where testing was needed, and providing counselling to patients on the use of these tests.^26^ To further substantiate pharmacists’ value in PGx provision, the American Society of Health Systems Pharmacists highlighted the importance of their role in leading international multidisciplinary teams for the integration of PGx services and the development of clinical practice guidelines. Additionally, PGx programs led by pharmacists have already been initiated in various settings.^60^,^64^ Therefore, in addition to physicians, the provision of PGx educational and training programs directed to pharmacists in the MENA region is essential considering their promising potential in PGx implementation.

Another important finding that emerged during the interviews was related to ethical dilemmas of using PGx testing. Participants have identified obtaining informed consent and ensuring transparency when delivering information as the most important aspects that need to be considered before testing their patients. These findings are in line with recommendations issued by the International Society of Psychiatric Genetics (ISPG) guidelines. They entailed that patients should be fully informed about the potential benefits and limitations of PGx testing, and that their consent should be obtained prior to testing. Patients should also be informed about the potential implications of test results for their treatment and any potential genetic risks that may be associated with the process. They also highlighted the importance of involving genetic counselors or other specialists in the process, as necessary, to ensure appropriate interpretation of test results.^65^ This is particularly important in the case of interpreting results for genes that may potentially carry disease risk implications. Multiple guidelines have been developed to regulate the informed consent process due to the potential risks that these tests impose.^66^ Although PGx-guided treatments require a genetic test, it should not be regarded as a threat for patients. Studies have highlighted key aspects to minimize any possible misconception or threats when discussing genetic testing, which should include: an explanation of the purpose of these tests, the procedures to be followed, and the expected outcomes to patients.^67^,^68^ Due to the variable options of genetic tests available, there is uncertainty around the consenting procedures that should be followed. Therefore, in order to overcome this issue and maintain patients’ rights, it is important to establish a set of standardized information to be disclosed to patients at the time of obtaining the consent. It is also recommended that instead of overloading patients with information on the risks and benefits of each gene included in the panel, this information should be further simplified and presented in a form of handouts that focus on certain themes in a “tiered and binned” approach. This approach is expected to support the informed decision process by providing data on various levels depending on each patients’ information needs and assist HCPs in moderating counselling sessions.^69^

## Strengths

To our knowledge, this was the first qualitative study aiming at improving the implementation of PGx testing in psychiatry in the MENA region through assessing perceptions, attitudes and preparedness of psychiatrists practicing within this region regarding this approach. It is worth noting that the study involved at least one participant from each of the GCC countries, which accounted for 66% of the total number of participants. This high participation from GCC countries also reflected good awareness of HCPs from this region regarding PGx testing and highlighted their high interest in research and medical advances. Furthermore, the study involved an acceptable number of participants from diverse countries within the region and the number included was adequate to reach saturation of data. Moreover, the qualitative nature of the study allowed for a comprehensive and deeper exploration of contexts underlying providers’ thoughts regarding PGx, which helped identify modifiable factors to be addressed in future implementation strategies. Finally, the use of inductive thematic analysis allowed for a more flexible and open-ended analysis of data, generated new insights, captured diverse perspectives, and enhanced validity of our findings.^70^

## Limitations

Some limitations might have influenced the obtained results. Firstly, the use of qualitative studies might possess some limitations as the responses provided were more subjective than objective. Moreover, the inclusion of physicians specialized in psychiatry only might have impacted the generalizability of the results since a PGx multidisciplinary team is expected to include a nurse, a geneticist, a pharmacist, and a genetic counsellor to facilitate the integration of PGx into clinical care.^71^ However, we selected our study population from physicians since in the majority of countries of the MENA region, prescribers lead health teams and are responsible for prescribing, ordering, and interpreting clinical tests for making clinical decisions.^28^ Additionally, the inclusion of psychiatrists could have affected generalizability in another way, as non-specialists in primary care could also prescribe psychiatric medications, hence assessing their views on PGx application is important.

Furthermore, we aimed initially at recruiting one participant from each country of the MENA region. We could not aim for a higher number due to the difficulties faced when recruiting participants from distant countries. Therefore, this data could serve as the basis for a more comprehensive evaluation involving different research designs, such as quantitative methods to validate our findings. Moreover, interviewer bias as well as leading questions bias could serve as a potential limitation for this study as it is expected that interviewers’ own beliefs and perceptions might influence the direction of the interview and the way the questions were being asked. However, to avoid the effect of these factors, the semi-structured design of questions allowed for open-ended answers and gave participants the opportunity to express their thoughts without interviewer’s intervention. In addition to that, all interviews were conducted by the same researcher, thus the risk of this bias was likely minimal.

## Conclusion

Results of this study highlight the interest of psychiatrists practicing within the MENA region in implementing PGx testing when managing people with depression and other psychiatric disorders. However, it is also important to recognize that this cannot be achieved unless more supporting nationwide strategies are implemented within health systems. As cost was viewed as a prominent barrier, enhancing the affordability of PGx testing is of paramount importance and it possibly would be best addressed through the public health sector. Additionally, this study provides preliminary findings that needs to be further confirmed through future research which entails conducting quantitative research to aid in understanding the level of HCPs knowledge, readiness and attitudes at a more universal level that would allow for a better generalizability of data.
